# *KRAS* mutation‐independent downregulation of MAPK/PI3K signaling in colorectal cancer

**DOI:** 10.1002/1878-0261.13163

**Published:** 2022-01-01

**Authors:** Kuen Kuen Lam, Choong Leong Tang, Emile Tan, Siew Heng Wong, Peh Yean Cheah

**Affiliations:** ^1^ Department of Colorectal Surgery Singapore General Hospital Singapore; ^2^ JW Bioscience Pte Ltd. Singapore Singapore; ^3^ Saw Swee Hock School of Public Health National University of Singapore Singapore; ^4^ Duke‐NUS Medical School National University of Singapore Singapore

**Keywords:** colorectal tumorigenesis, CPTAC, KRAS signaling, MAPK/PI3K, SOX9, TCGA

## Abstract

*KRAS* is a gatekeeper gene in human colorectal tumorigenesis. KRAS is ‘undruggable’; hence, efforts have been diverted to inhibit downstream RAF/MEK/ERK and PI3K/Akt signaling. Nevertheless, none of these inhibitors has progressed to clinical use despite extensive trials. We examined levels of phospho‐ERK1/2(T202/Y204) and phospho‐Akt1/2/3(S473) in human colorectal tumor compared to matched mucosa with semi‐quantitative near‐infrared western blot and confocal fluorescence immunohistochemistry imaging. Surprisingly, 75.5% (25/33) of tumors had lower or equivalent phospho‐ERK1/2 and 96.9% (31/32) of tumors had lower phospho‐Akt1/2/3 compared to matched mucosa, irrespective of *KRAS* mutation status. In contrast, we discovered *KRAS*‐dependent SOX9 upregulation in 28 of the 31 (90.3%) tumors. These observations were substantiated by analysis of the public domain transcriptomics The Cancer Genome Atlas (TCGA) and NCBI Gene Expression Omnibus (GEO) datasets and proteomics Clinical Proteomic Tumor Analysis Consortium (CPTAC) dataset. These data suggest that RAF/MEK/ERK and PI3K/Akt signaling are unlikely to be activated in most human colorectal cancer.

## Graphical abstract

Phospho‐ERK1/2 and phospho‐Akt1/2/3 are downstream effectors of KRAS signaling frequently used as endpoints for drug testing. We showed that phospho‐ERK1/2 and phospho‐Akt1/2/3 are not upregulated in colorectal cancer (CRC) tumors compared to matched mucosa, regardless of *KRAS* mutation status. Instead, SOX9 is upregulated in a *KRAS*‐dependent manner and thus may be a more suitable effector of KRAS signaling in CRC.

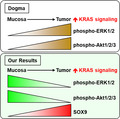

**Abbreviations**
ACF: aberrant crypt foci
ACTB: beta‐actin
Akt: protein kinase B
ANOVA: analysis of variance
Apc: adenomatous polyposis coli
BCA: bicinchoninic acid
COAD: colon adenocarcinoma
CPTAC: clinical proteomic tumor analysis consortium
CRC: colorectal cancer
EGFR: epidermal growth factor receptor
ERK: extracellular signal‐regulated kinases
GDC: genomic data commons
GEO: gene expression omnibus
GO:BP: gene ontology:biological processes
GTP: guanosine triphosphate
I.I: integrated intensity
IHC: immunohistochemistry
IkBα: inhibitor of nuclear factor kappa B
kDa: kilo Dalton
KEGG: kyoto encyclopedia of genes and genomes
KRAS: kirsten rat sarcoma virus
MAPK: mitogen‐activated protein kinase
MCMs: minichromosome maintenance protein complexes
MDC1: mediator of DNA damage checkpoint 1
MEK: MAPK/ERK Kinase
NC: normal crypt
NCBI: National Center for Biotechnology Information
NF‐kB: nuclear factor kappa‐light‐chain‐enhancer of activated B cells
NI‐WB: near‐infrared western blot
PBS: phosphate buffered saline
PDAC: pancreatic ductal adenocarcinoma
PI3K: phosphoinositide 3‐kinase
Raf: rapidly accelerated fibrosarcoma
Ras: rat sarcoma virus
SDS/PAGE: sodium dodecyl sulfate/polyacrylamide gel electrophoresis
SOX9: sex‐determining region Y‐box transcription factor 9
TAK1: transforming growth factorbetaactivated kinase 1
TCGA: the cancer genome atlas
vs: versus
Wnt: wingless and Int‐1
WT: wild‐type

## 1 Introduction

Colorectal cancer (CRC) is the world’s fourth most deadly cancer with almost 900 000 deaths annually [1]. CRC is usually asymptomatic in early stages and hence frequently diagnosed at the late stages [1]. The 5‐year age‐standardized observed survival is only 60% and 10% for lymph node involved Stage III and distal organ metastasized Stage IV CRC, respectively [2]. Treatment options for metastasized CRC include chemotherapy, targeted therapy, and immunotherapy. While immunotherapy is a promising development in cancer therapy, majority (~90%) of CRC are microsatellite stable and not amenable to immunotherapy. Currently, the success rate of first‐line chemotherapy of 5‐Fluorouracil and oxaliplatin for advanced or metastatic CRC is less than 30% [3, 4]. Patients who do not respond to this first‐line therapy and have wild‐type (WT) *KRAS* gene are sometimes given the anti‐EGFR (epidermal growth factor receptor)‐targeted therapy Cetuximab or Panitumumab. Nevertheless, anti‐EGFR treatment is only limited to CRC with WT KRAS, the predominant form of Ras in CRC [5]. KRAS is a central activator of EGFR signaling. Upon EGFR stimulation, it undergoes activation via GTP exchange mediated by guanine nucleotide exchange factors. KRAS oncogenic mutants are resistant to inactivation by GTPase‐activating proteins, bypassing the need for continual activation by EGFR. Approximately 50% of CRC harbors KRAS oncogenic mutations [6]. Still, patients who receive anti‐EGFR treatment eventually develop resistance due to therapy‐induced *KRAS* oncogenic mutations driven by natural selection [7, 8]. Oncogenic RAS has been shown to be essential for tumor maintenance and KRAS mutation in CRC is associated with metastasis and poor prognosis [9, 10, 11, 12].

Given its important role in CRC progression, KRAS is an obvious therapeutic target. Unfortunately, direct inhibition of KRAS is exceptionally challenging as it is not receptive to inhibitor docking [13, 14]. To date, the only approved direct KRAS inhibitor is Sotorasib (AMG 510) which specifically targets KRAS G12C in non‐small‐cell lung carcinoma by forming a covalent bond with the cysteine [15]. Nevertheless, KRAS G12C mutations are rare in CRC. Alternative strategies like inhibition of farnesyl transferase which prevents KRAS C‐terminal prenylation, required for inner plasma membrane localization where KRAS functions, were unsuccessful as farnesyl transferase are functionally replaced by geranylgeranyl transferase [16, 17]. Efforts were then diverted to inhibit KRAS downstream targets Raf/MEK/ERK, also known as mitogen‐activated protein kinase (MAPK) and PI3K/Akt [18, 19]. Despite extensive efforts in research and clinical trials, none of the Raf/MEK/ERK and PI3K/Akt inhibitors have progressed to clinical use for CRC. In fact, to date, only one clinical trial (NCT02788279) has progressed past Phase 2 (reviewed by Xie et al. [20]).

The discrepancy of the MAPK and PI3K inhibitor preclinical studies using CRC cell lines and clinical trials outcome led us to question whether MAPK/PI3K signaling is commonly upregulated in human CRC with *KRAS* mutations. As most preclinical trial research of MAPK/PI3K signaling was performed on CRC cell lines and mouse models, they may not emulate the condition in human CRC. Most importantly, we are interested in the extent of activation of MAPK/PI3K signaling in human CRC tumor compared to the corresponding morphologically normal matched mucosa.

## 2 Methods

### 2.1 Study samples

Tumor and matched mucosa samples from resected colon of CRC patients were collected between 2007 and 2017, snap‐frozen in liquid nitrogen, and stored in −80 °C in the Department of Colorectal Surgery Tissue Repository, Singapore General Hospital, with written informed consent. Samples used in this study had paired morphologically normal mucosa at least 10 cm away from the tumor. A total of 33 pairs of CRC tumor and matched mucosa tissues were used, which includes tumor with *KRAS* WT (*n* = 8), G12D (*n* = 8), G12V (*n* = 9), G13D (*n* = 5), and others (*n* = 3). The clinico‐pathological characteristics of the samples are summarized in Table S1. The study methodologies conform to the standards set by the Declaration of Helsinki and are approved by the SingHealth Centralised Institutional Review Board (CIRB project number 2018/2837).

### 2.2 PCR amplification and sequencing of *KRAS* coding sequences

*KRAS* Exons 2, 3, 4 harboring the coding sequence were PCR amplified from patient genomic DNA using GoTaq® Flexi DNA Polymerase Kit (Promega, WI, USA). The PCR products were purified by isopropanol precipitation. Sequencing was performed with Applied Biosystems (MA, USA) BigDye™ Terminator v3.1 Cycle Sequencing Kit using the PCR products as templates. The reaction was purified by ethanol precipitation and resuspended in Hi‐Di™ Formamide and then separated by capillary electrophoresis in 3500 Genetic Analyzer (Applied Biosystems). The output sequencing results were visualized on Chromas v2.6.4 (Technelysium Pty Ltd, South Brisbane, Australia) as chromatograms and visually inspected for mutations. Both PCR and Sanger sequencing reaction were performed on Biometra (Analytik Jena AG, Jena, Germany) UNOII or GeneAmp 9700 (Applied Biosystems) Thermocyclers. All primers are listed in Table S2.

### 2.3 Protein extraction and concentration reading

Tumor tissues were enriched for neoplastic cells (at least 90%) by macro‐dissection while mucosa were used directly after slicing. For protein extraction, tissues were lysed in Tissue Protein Extraction Reagent (T‐PER™) supplemented with Halt™ protease and phosphatase inhibitor cocktail (Thermo Fisher Scientific, MA, USA), homogenized with a micro pestle driven by a cordless pestle motor. Protein concentrations were taken with the Pierce™ BCA Protein Assay Kit (Thermo Fisher Scientific) with BSA as standards.

### 2.4 SDS/PAGE and NI‐WB

For SDS/PAGE, 10 µg of protein lysates were loaded per well for phospho‐ERK1/2 / total ERK1/2 and SOX9 / ACTB and 20 µg of protein lysates per well for phospho‐Akt1/2/3 / pan‐Akt1/2/3. The loaded samples were separated by electrophoresis using a 10% SDS/PAGE gel in 1X Tris/Glycine/SDS Buffer, ran at 30V, 40 min, then 100 V, 2 h. The SDS/PAGE was transferred to nitrocellulose membrane with Bio‐Rad (CA, USA) Transblot using a preset program. After transfer, the membranes were briefly rinsed in MilliQ filtered water and air‐dried in fume hood for 1 h. Thereafter, the membrane was rehydrated in PBS for 2 min and then incubated in Intercept Blocking Buffer (LI‐COR Biosciences, NE, USA) at room temperature for 2 h with gentle orbital shaking (80 r.p.m.). All primary antibodies were diluted in Intercept Blocking Buffer with 0.2% Tween 20. The membranes were incubated with the diluted antibodies at 4–8 °C overnight (~ 16 h) with gentle orbital shaking. For SOX9 / ACTB NI‐WB, the membrane was incubated with only diluted anti‐SOX9 antibody overnight and then incubated in anti‐ACTB antibody in room temperature for 2 h with gentle orbital shaking (80 r.p.m.). After primary antibody incubation, the membranes were washed with PBS with 0.1% Tween 20 for 5 min, total 3 times. The membranes were then incubated in secondary antibodies diluted in Intercept Blocking Buffer with 0.2% Tween 20 at room temperature for 1 h with gentle orbital shaking (80 r.p.m.). The membranes were washed then with PBS with 0.1% Tween 20 for 5 min, total 3 times, then in PBS 2 times before imaging. The membranes were imaged with the Odyssey imaging system (LI‐COR Biosciences). Integrated Intensity (I.I) K counts values with lane background subtraction, representing WB band intensity, correlating with protein abundance, was calculated by the Odyssey software v3 (LI‐COR Biosciences). All primary and secondary antibodies and their corresponding dilutions are listed in Table S3.

### 2.5 Confocal fluorescence immunohistochemistry

Cryosections of 8 µm thickness were prepared from 8 out of the 33 pairs of fresh‐frozen CRC tumor and matched mucosa tissues used in NI‐WB. Briefly, the cryosections were methanol‐fixed and permeabilized. The fixed cryosections were blocked in blocking buffer [5% goat serum, 5% fetal bovine serum, 3% BSA in PBS with calcium and magnesium] and subsequently incubated with Rabbit anti‐phospho‐ERK1/2 (Thr202/Tyr204) (Cell Signaling Technology, Danvers, MA, USA, cat# 4370) or mouse anti‐SOX9 (Sigma‐Aldrich, MO, USA, cat# AMAB90795), both diluted 1 : 200. After washing, the cryosections were probed with AlexaFluor 555 goat anti‐mouse IgG or AlexaFluor 488 goat anti‐rabbit IgG (Cell Signaling Technology cat# 4409 and 4412, respectively), both diluted 1 : 1000. The immunostained cryosections were imaged with Nikon A1 confocal microscope (Nikon, Tokyo, Japan).

### 2.6 Statistics and graph plotting

Comparisons between tumor and matched mucosa were performed with paired *t*‐test. Comparisons between tumor of different KRAS and BRAF mutations were performed with unpaired *t*‐test with Welch's correction. One‐way ANOVA was used to test if any group (i.e., KRAS or BRAF mutation) has a significant difference from others. Linear regression was performed to test the correlation between tumor SOX9 and phospho‐ERK1/2 levels from NI‐WB. The cutoff *P*‐value is *P* < 0.05 for all analyses. All statistics and graph plotting were performed on the graphpad prism v9.1.2 software (graphpad Software, CA, USA).

### 2.7 Retrieval and analysis of public domain phosphoproteome, proteome, and transcriptome data

Proteome and phosphoproteome final data metrics were retrieved from National Cancer Institute’s Clinical Proteomic Tumor Analysis Consortium (CPTAC) Colon Adenocarcinoma (COAD) dataset via the LinkedOmics portal (http://linkedomics.org/data_download/CPTAC‐COAD/) [21]. The dataset consists of 96 pairs of tumor and matched mucosa (details in Table S4). The normalized Log_2_FC (fold‐change) of phospho‐ERK1/2 (MAPK3_Y204 and MAPK1_Y187) and SOX9 of Tumor vs Normal (i.e., mucosa) were retrieved from the phosphoproteome and proteome dataset, respectively, and the samples were matched to their corresponding KRAS and BRAF mutation status. Phospho‐Akt1/2/3(S473) data were not available from CPTAC. CRC tumor SOX9 mRNA levels were from RNA sequencing data in RSEM (RNA‐Seq by Expectation‐Maximization) values batch normalized from Illumina RNASeqV2 data were retrieved via cBioPortal (https://www.cbioportal.org/) [22, 23] from the TCGA Pan Cancer Atlas database.

Transcriptome data were downloaded from The Cancer Genome Atlas (TCGA) Genomics Data Commons (GDC) portal (https://portal.gdc.cancer.gov/) and NCBI Gene Expression Omnibus (GEO) data repository (https://www.ncbi.nlm.nih.gov/geo/), series GSE95132 [24]. Transcriptome retrieved from TCGA were from 35 pairs of CRC tumor with matched mucosa; tumors were microsatellite‐stable with known KRAS mutation status (24 KRAS WT and 11 KRAS mutant) (details in Table S4) [25]. GSE95132 consists of 10 pairs of CRC tumor and matched mucosa, and 5 pairs of aberrant crypt foci (ACF) and matched normal crypts (NCs), all tumors and ACFs harbor *KRAS* somatic mutations (personal communications with Prof Daniel Rosenberg) [24]. Transcriptome data downloaded from TCGA GDC portal were htseq‐count files while data from NCBI GEO GSE95132 were raw RNA sequencing fastq files. Both TCGA transcriptome htseq count files and GSE95132 raw RNA sequencing reads were analyzed on the Galaxy server (https://usegalaxy.org/) [26]. In brief, htseq count files from TCGA were directly analyzed with DESeq2 module to generate a list of differentially expressed genes between tumor and matched mucosa [27]. Raw sequencing files from GSE95132 were first mapped to the human genome build hg38 with HISAT2 module and then htseq count reads generated with htseq‐count module [28, 29]. Similarly, differentially expressed genes between tumor and matched mucosa and aberrant crypt foci (ACF) and normal crypts (NCs) are generated with DESeq2 module from htseq‐count files which outputs log_2_FC (fold‐change) of sample vs control with *P*‐value adjusted for multiple testing with the Benjamini–Hochberg procedure [27]. Significantly upregulated genes in CRC tumor vs mucosa, and ACF vs NCs were fed into DAVID Annotation Tool (https://david.abcc.ncifcrf.gov/summary.jsp) [30, 31] and g:Profiler (https://biit.cs.ut.ee/gprofiler/) [32, 33]. The number of input and mapped differentially expressed genes for DAVID and g:Profiler analysis are summarized in Table S5. Output consists of Gene ontology biological process (GO : BP), Kyoto Encyclopedia of Genes and Genomes (KEGG) pathway and REACTOME pathway terms associated with the input genes and the corresponding *P*‐values indicating significance of association. Output from DAVID uses Modified Fisher Exact adjusted *P*‐value [30, 31] and output from g:Profiler uses tailor‐made algorithm g:SCS adjusted *P*‐value [32, 33].

## 3 Results

### 3.1 Lower phospho‐ERK1/2 levels detected in tumor compared to matched mucosa

**Fig. 1:**
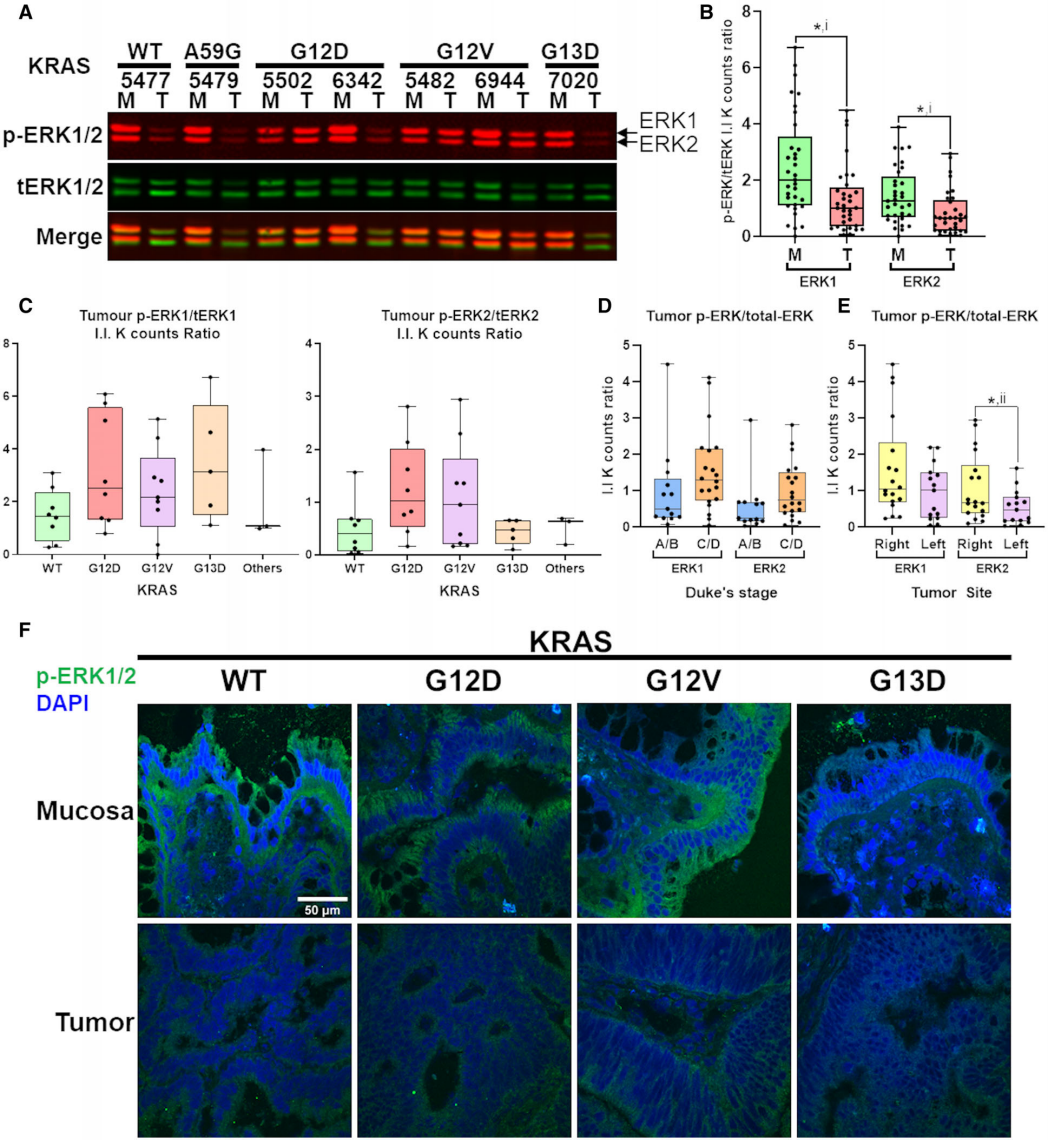
Downregulation of phospho‐ERK1/2 in CRC tumor compared to matched mucosa. A representative NI‐WB of phospho‐ERK1/2 and total‐ERK1/2 for 7 pairs of CRC tumor and matched mucosa with tumor *KRAS* mutation status indicated on top (A). Box and whiskers plot of NI‐WB phospho‐ERK/total‐ERK I.I K counts ratio for ERK1 and ERK2 of pooled mucosa and tumor (B), of tumor grouped by *KRAS* mutation (C), Duke’s stage (A/B early stage; C/D advanced stage) (D) and tumor site (right‐colon; left‐colon) (E). Representative confocal images of phospho‐ERK1/2 (green) IHC staining with nuclear DAPI (blue) counterstain of CRC‐matched mucosa and tumor cryosections for each *KRAS* mutation status, scale bar = 50 µm in top left image is applicable to all images (F). *two‐tailed *t*‐test *P* < 0.05; i, paired *t*‐test; ii, unpaired *t*‐test with Welch’s correction. M, mucosa; p‐ERK1/2, phospho‐ERK1/2; T, tumor; tERK, total‐ERK. Both NI‐WB (A‐D) and IHC (F) were performed on biological replicates (*n* = 33 and *n* = 8, respectively).

Despite extensive efforts in research and clinical trials, none of the Raf/MEK/ERK and PI3K/Akt inhibitors have progressed to clinical use for CRC [20]. It is worth noting that most of the earlier studies were performed using KRAS oncogenic mutant cancer CRC cell lines. We hypothesized that the failure in targeting the MAPK (Raf/MEK/ERK) and PI3K/Akt pathways in CRC could be likely due to the fact that both pathways are not activated in primary tumors in CRC. To determine the activation states of the MAPK pathway in primary tumor versus (vs) match mucosa, we performed NI‐WB to measure the levels of phospho‐ERK1/2(T202/Y204, T285/Y187) in primary tumors vs matched mucosa resected from CRC patients. The protein levels were quantified by calculating the I.I K counts of the 44/42 kDa phospho‐ERK1/2 and total‐ERK1/2 protein bands. The phospho‐ERK1/2 protein levels were internally normalized to total‐ERK1/2 protein levels within each sample. Subsequent mention of phospho‐ERK1/2 levels refer to protein levels normalized to total‐ERK1/2. NI‐WB revealed that 25 out of 33 (75.8%) of CRC tumor had lower or equivalent phospho‐ERK1/2 compared to their matched mucosa tissues (Fig. 1A,B; Fig. S1). The phospho‐ERK1 and phospho‐ERK2 levels in tumor are significantly lower than matched mucosa (Fig. 1B). Also, tumor phospho‐ERK1 and phospho‐ERK2 levels were not significantly different between *KRAS* mutation status (one‐way ANOVA, *P* > 0.05) (Fig. 1C). This indicates that the extent of decrease in tumor phospho‐ERK1/2 expression level is not associated with *KRAS* mutation status. The tumor phospho‐ERK1/2 levels were also not significantly different between Duke’s stage A/B and C/D tumors (unpaired two‐tailed *t*‐test) (Fig. 1D). There is no significant difference in phospho‐ERK1 levels between right‐ and left‐sided tumors, but significantly higher phospho‐ERK2 in right‐sided tumor (Fig. 1E). Confocal fluorescence immunohistochemistry (IHC) study of phospho‐ERK1/2 in human CRC‐matched mucosa and tumor cryosections show that expression is relatively high in mucosa epithelium while low in tumor (Fig. 1F).

### 3.2 PI3K/Akt pathway activation is similarly not detected in CRC tumor by NI‐WB

**Fig. 2:**
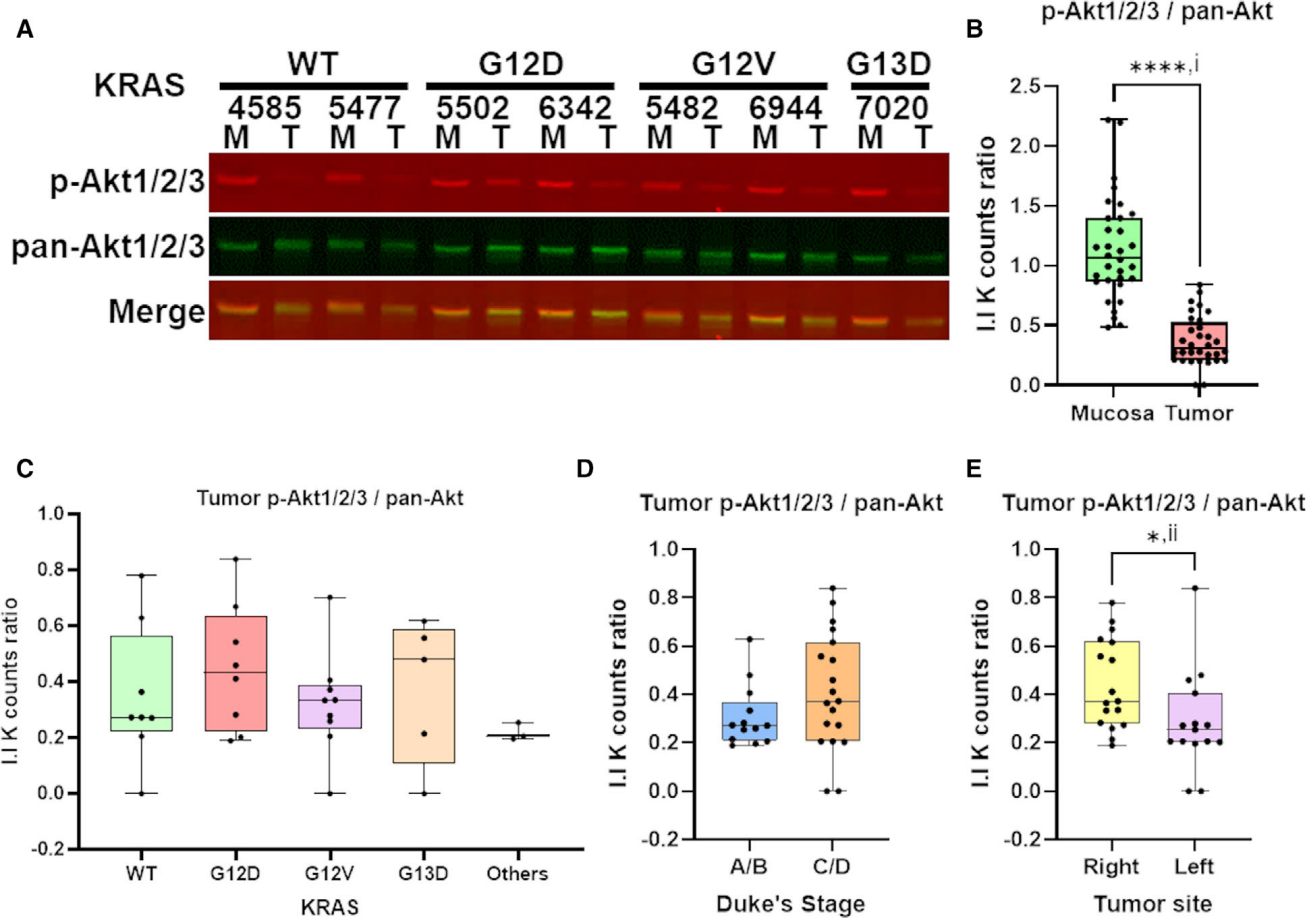
Downregulation of phospho‐Akt1/2/3 in CRC tumor compared to matched mucosa. A representative NI‐WB of phospho‐Akt1/2/3 and pan‐Akt for 7 pairs of CRC tumor and matched mucosa with tumor *KRAS* mutation status indicated on top (A). Box and whiskers plot of NI‐WB phospho‐Akt1/2/3 / pan‐Akt I.I K counts ratio of pooled mucosa and tumor (B), of tumor grouped by *KRAS* mutation (C), Duke’s stage (A/B early stage; C/D advanced stage) (D) and tumor site (right‐colon; left‐colon) (E). *two‐tailed *t*‐test *P* < 0.05; ****two‐tailed *t*‐test *P* < 0.0001; i, paired *t*‐test; ii, unpaired *t*‐test with Welch’s correction. M, mucosa; p‐Akt1/2/3, phospho‐Akt1/2/3; T, tumor. NI‐WB (A‐D) was performed on biological replicates (*n* = 32).

KRAS mutation is reported to activate PI3K/Akt signaling; it is thus interesting to determine whether the PI3K/Akt pathway is similarly perturbed in primary tumor of CRC. We performed NI‐WB of phospho‐Akt1/2/3(S473) and pan‐Akt1/2/3 on the same 32 pairs of human CRC tumors and matched mucosa; 1 pair was omitted due to protein degradation. The phospho‐Akt1/2/3 protein levels were internally normalized to pan‐Akt levels within each sample. NI‐WB revealed that 31/32 (96.9%) of tumor has lower phospho‐Akt1/2/3 compared to matched mucosa (Fig. 2A,B; Fig. S2). The phospho‐Akt1/2/3 levels in tumor are significantly lower than matched mucosa (Fig. 2B). Also, the tumor phospho‐Akt1/2/3 levels are not significantly different between different KRAS mutation status (one‐way ANOVA, *P* > 0.05) (Fig. 2C). This indicates that decrease in tumor phospho‐Akt1/2/3 levels is not dependent on KRAS mutation status. The tumor phospho‐Akt1/2/3 levels are also not significantly different between Duke’s stage A/B and C/D tumors (unpaired two‐tailed *t*‐test) (Fig. 2D). Tumor phospho‐Akt1/2/3 is significantly higher in right‐sided compared to left‐sided tumor (Fig. 2E).

### 3.3 SOX9 protein is upregulated in most human CRC tumors

**Fig. 3:**
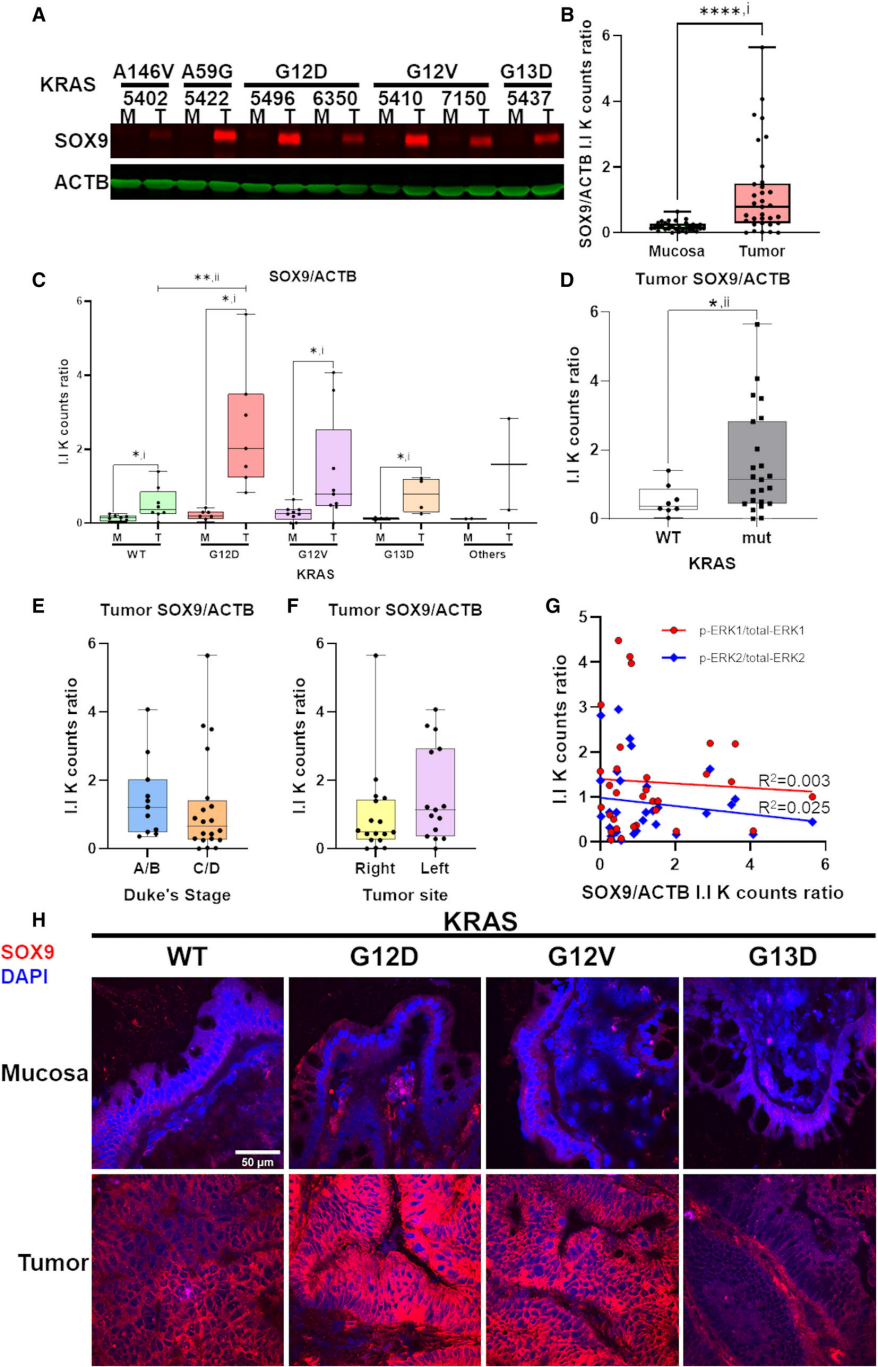
KRAS mutation‐dependent upregulation of SOX9 in CRC tumor compared to matched mucosa. A representative NI‐WB of SOX9 and ACTB for 7 pairs of CRC tumor and matched mucosa (A). Box and whiskers plot of SOX9/ACTB I.I K counts ratio of mucosa and tumor pairs from NI‐WB (*n* = 31) (B). Box and whiskers plot of NI‐WB SOX9/ACTB I.I K counts ratio of mucosa and tumor tissues grouped by tumor *KRAS* mutation status (C), of *KRAS* WT and pooled mutant tumor (D), of tumor grouped by Duke’s stage (A/B early stage; C/D advanced stage) phospho‐ERK (E) or tumor site (right‐colon; left‐colon) (F). Linear regression of NI‐WB tumor SOX9/ACTB with phospho‐ERK1/total‐ERK1 or phospho‐ERK2/total‐ERK2 I.I K counts ratio (G). Representative confocal images of IHC of SOX9 (red) with nuclear DAPI (blue) counterstain of CRC‐matched mucosa and tumor cryosections for each *KRAS* mutation status, scale bar = 50 µm in top left image is applicable to all images (H). *one‐tailed *t*‐test *P* < 0.05; **two‐tailed *t*‐test *P* < 0.05; ****two‐tailed *t*‐test *P* < 0.0001. i, paired *t*‐test; ii, unpaired *t*‐test with Welch’s correction. M, mucosa; mut, mutant; T, tumor. Both NI‐WB (A–D) and IHC (H) were performed on biological replicates (*n* = 31 and *n* = 8, respectively).

It is highly possible that other KRAS effectors in CRC could be affected in the primary tumors since our results showed that MAPK and PI3K/Akt pathways were not found to be upregulated in the primary tumors in CRC. One of the KRAS effectors of interest is SOX9. SOX9 had been previously reported to be part of the EGFR/ERK/SOX9 cascade [34]. Thus, it is interesting to determine whether downregulation of phospho‐ERK1/2 observed in this study affects the expression of SOX9 in primary tumors in CRC. We investigated SOX9 protein levels in the same 33 pairs of human CRC tumors and matched mucosa, 2 tumor samples were omitted due to protein degradation observed in ACTB NI‐WB. Unexpectedly, 28 out of 31 (90.3%) tumors have higher SOX9 protein levels (normalized to ACTB) than their matched mucosa (Fig. 3A,B; Fig. S3). SOX9 is significantly higher in pooled tumor than matched mucosa (Fig. 3B). SOX9 was found to be significantly upregulated in KRAS WT and mutant tumors compared to matched mucosa (Fig. 3C). Particularly, KRAS G12D had significantly higher tumor SOX9 than KRAS WT (Fig. 3C). Pooled KRAS mutant tumors SOX9 protein levels are also significantly higher than WT (Fig. 3D). The protein levels of tumor SOX9 are not significantly different between Duke’s stage A/B and C/D (Fig. 3E) or between right‐ and left‐sided colon (Fig. 3F). Linear regression analysis showed no significant correlation between tumor SOX9 and phospho‐ERK1/2 levels (Fig. 3G). Confocal fluorescence IHC study showed higher SOX9 staining in tumor compared to matched mucosa cryosections, especially in tumor harboring KRAS G12D and G12V mutations (Fig. 3H).

## 4 Discussion

In our study, we quantified phospho‐ERK1/2 and phospho‐Akt1/2/3 protein levels internally normalized to total‐ERK1/2 and pan‐Akt, respectively, using the LI‐COR system of NI‐WB. The NI‐WB results showed that the activation states of MAPK and PI3K/Akt is relatively lower in most CRC primary tumors (Fig. 1A,B, Fig. S1 and Fig. 2A,B, Fig. S2), regardless of KRAS oncogenic mutation status. Similarly, confocal fluorescence IHC study also showed lower phospho‐ERK1/2 in primary tumor as compared to matched mucosa (Fig. 1F). Interestingly, analysis performed on the phosphoproteomics data provided by the CPTAC‐COAD dataset [21] also showed that phospho‐ERK1(Y204) and phospho‐ERK2(Y187) were significantly lower in tumor than matched mucosa [log_2_FC (fold‐change) < 0] regardless of the *KRAS* mutation status (one‐way ANOVA, *P* < 0.05) (Fig. S4). This further supported our results showing that the presence of KRAS oncogenic mutation did not induce upregulation of activated phospho‐ERK1/2 in CRC primary tumors.

Our results were in agreement with previous studies reporting decreased MAPK activity of human CRC tumor compared with matched mucosa [35, 36, 37, 38]. Hoshino et al. [39] reported increased MAPK activation in only 7/34 human colon tumors compared to nontumorous tissues from the same individuals. In Haigis et al. [40], all 18 human primary CRC tissues did not have positive phospho‐ERK1/2 IHC staining while 4 of 18 adjacent mucosa had positive staining. Gulmann et al. [41] reported significantly lower phospho‐ERK1/2 in human CRC tumor epithelium compared to matched mucosa epithelium from IHC and western blot experiments. Yeh et al. [42] also reported significantly lower nuclear and cytoplasmic phospho‐ERK1/2 in human CRC tumor epithelium compared to matched mucosa epithelium from IHC of 190 pairs of samples. For studies without mucosa comparison, Sakakura et al. [43] detected MAPK activation in only 4 out of 21 (18%) advanced colon cancer. Schmitz et al. [44] reported positive phospho‐ERK1/2 IHC staining in only 20 out of 115 (14.8%) human CRC tumors. Altogether, MAPK signaling is rarely activated in CRC tumor. Nevertheless, four studies reported higher tumor MAPK activity compared to matched mucosa [45, 46, 47, 48]. While these studies addressed the state of MAPK activation in CRC tumor, there was no association drawn with KRAS mutation status. In our study, we included a substantial number of tumors with common *KRAS* mutations (i.e., G12D, G12V, G13D); henceforth, revealing that tumor phospho‐ERK1/2 levels are not dependent on *KRAS* oncogenic mutations (Fig. 1C). In agreement, Perkins et al. [49] observed no significant difference in phospho‐ERK1/2 and phospho‐Akt levels between KRAS WT (*n* = 23) and mutant (*n* = 19) CRC tumors, but there was no comparison with mucosa. We only selected samples with matched mucosa at least 10 cm from tumor site to ensure that the mucosa are nontransforming and outside of tumor paracrine influence, providing a suitable basal control. Interestingly, the right‐sided tumors have significantly higher phospho‐ERK2 (Fig. 1E) and phospho‐Akt1/2/3 (Fig. 2E) than left‐sided tumors, re‐affirming the different cancer biology between right‐ and left‐sided colon [50].

The NI‐WB using the LI‐COR system allows accurate relative quantification of phospho‐ERK1/2 and phospho‐Akt1/2/3 levels owing to this system’s large dynamic range compared to methods used in previous studies. NI‐WB is also more quantitative as phospho‐ERK1/2 and phospho‐Akt1/2/3 are internally normalized to total‐ERK1/2 and pan‐Akt, respectively. Importantly, the NI‐WB assay revealed high mucosa expression of phospho‐ERK1/2 in the matched mucosa (Fig. 1A) suggesting a role in normal colonic homeostasis; targeting phospho‐ERK1/2 may thus not be ideal. MAPK and PI3K signaling pathways are crucial in maintaining integrity of tight junctions in normal colonic epithelial cells [51, 52]. Thus, high mucosa expression of phospho‐ERK1/2 and phospho‐Akt1/2/3 is required to maintain integrity (organization) of differentiated epithelial cells in the crypts (Fig. S5, panels A and C). Interestingly, even in the tumors, expression of phospho‐ERK1/2 plays a role in determining the organization of crypt’s epithelial cells. Higher numbers of collapsed/deformed crypts were observed in primary tumor with downregulated phospho‐ERK1/2 expression (Fig. S5, panel B) as compared to primary tumors with upregulated phospho‐ERK1/2 expression (Fig. S5, panel D).

While several studies have reported high MAPK activity in CRC cancer cells with KRAS oncogenic mutations, most of them were performed on cell lines or mouse models. Cell lines are highly culture‐adapted and lack the microenvironment of the native tissue, hence unlikely to replicate biological properties of human CRC [53]. Moreover, several studies used phospho‐ERK1/2 downregulation as a biomarker to test candidate drug effectiveness on CRC cell lines without a noncancerous control for comparison of MAPK activation. A few studies which assayed MAPK activation in multiple CRC cell lines have vast variability between cell lines. In Hoshino et al. [39], only 7/17 CRC cell lines had constitutive MAPK activity. Kress et al. [54] described substantial variation of phospho‐ERK1/2 levels in 64 CRC cell lines, with 25 high, 18 moderate and 21 low to nondetectable phospho‐ERK1/2 levels. Mouse models of CRC have significant differences with human, as well as the method of CRC induction usually involves either chemical (i.e., 1,2‐dimethylhydrazine) treatment or pan‐colonic *Apc* knockout which is starkly different from human sporadic CRC tumorigenesis which likely arise from random mutation events of a single initiating cell [55, 56]. As most preclinical studies of drugs targeting MAPK signaling were performed on cell lines and mouse xenografts of cell lines, they may not be translated into clinical settings [57].

Our results raised the question of how activation of the MAPK/PI3K signaling pathways were perturbed in CRC primary tumors as compared to matched mucosa, regardless of the KRAS mutation status. To shine more light on this issue, bioinformatics analyses were performed on transcriptome datasets extracted from TCGA and NCBI GEO series GSE95132 [24]. Results from this analysis showed that upregulated genes in tumor vs mucosa and aberrant crypt foci vs adjacent normal crypts were not significantly associated with MAPK‐related and PI3K‐related Gene Ontology Biological Processes (GO : BP), KEGG, and REACTOME pathway terms (Tables S6A, S7A). On the other hand, downregulated genes in tumor from both the *KRAS* WT and mutant TCGA datasets were observed to be significantly associated with 3 MAPK‐related and 6 PI3K/Akt‐related GO : BP terms (Tables S6B, S7B). Downregulated genes from TCGA KRAS WT but not mutant was significantly associated with the KEGG term hsa04010 : MAPK signaling pathway (Table S6B). Analysis of these downregulated genes related to the MAPK and PI3K/Akt signaling pathways will shed light on how ERK1/2 and Akt1/2/3 were perturbed in CRC primary tumors independent of *KRAS* oncogenic mutants. Our hypothesis is that this may be related to downregulation of EGFR in CRC tumors as interrogating the CPTAC dataset showed significant *KRAS* mutation‐independent downregulation of EGFR in the tumors compared to the matched mucosa (*n* = 96 pairs; *P* < 0.0001). A recent study has also provided evidence that Wnt‐signaling could repress MAPK signaling in several mouse cancer models [58].

Notably, our NI‐WB results indicated that SOX9 protein is upregulated in the tumor compared to matched mucosa (Fig. 3A,B; Fig. S3), and KRAS G12D or pooled KRAS mutant had significantly higher SOX9 protein levels than WT tumors (Fig. 3C,D). Similarly, confocal fluorescence IHC showed higher SOX9 in tumor compared to matched mucosa, with stronger SOX9 immunostaining in KRAS G12D and G12V tumors (Fig. 3H). In agreement, SOX9 protein levels in the CPTAC‐COAD proteome dataset are significantly upregulated in tumors compared to matched mucosa, with significantly higher upregulation in KRAS G12V compared to WT (Fig. S6A) [21]. Analyses of the TCGA and GSE95132 paired tumor‐mucosa transcriptome datasets also revealed SOX9 to be significantly upregulated in tumor compared to matched mucosa (Table S8).

Furthermore, TCGA Pan Cancer Atlas dataset (*n* = 435) retrieved via cBioPortal exhibited significantly higher SOX9 mRNA levels in CRC tumors with KRAS mutations compared to KRAS WT (Fig. S6B) [22, 23]. This indicates that KRAS mutant CRC is associated with higher SOX9 upregulation, indicating possible activation by oncogenic KRAS signaling. On the other hand, we found no correlation between tumor phospho‐ERK1/2 and SOX9 protein levels, indicating that SOX9 is unlikely to be activated by phospho‐ERK1/2 via the EGFR‐ERK‐SOX9 axis as reported in urothelial cancer (Fig. 3G) [34]. Human Protein Atlas IHC also showed intense SOX9 antibody staining in the crypt epithelium of CRC tumor sections (https://www.proteinatlas.org) [59, 60, 61]. SOX9 is a member of the SOX family of transcription factors containing a high mobility group (HMG) DNA binding domain and a transactivation domain. SOX9 plays a role in several developmental and self‐renewal processes. Of note, it is upregulated by Wnt/β‐catenin signaling and promotes intestinal stem cell renewal. SOX9 is overexpressed in hepatocellular, breast, bladder, gastric, prostate, ovarian, pancreatic, and colorectal cancer [62, 63]. A TCGA study predicted SOX9 to be a possible CRC driver [64, 65]. In pancreatic ductal adenocarcinoma (PDAC), another KRAS‐driven cancer, SOX9 is a critical mediator of KRAS‐induced PDAC initiation of pancreatic acinar cells in mouse model [66]. SOX9 has also been implicated to be upregulated by oncogenic KRAS via activation of TAK1/IκBα/NF‐κB signaling in PDAC, in turn promoting PDAC by upregulation of minichromosome maintenance protein complexes (MCMs) and mediator of DNA damage checkpoint 1 (MDC1) [67]. Taken the evidences together, we propose that SOX9 could be a novel KRAS downstream effector and a potential therapeutic target but further research into its role in CRC is required.

## 5 Conclusions

In conclusion, while MAPK/PI3K signaling is widely accepted to be mitogenic, it is not activated in most CRC tumors regardless of KRAS mutation status. The widespread use of phospho‐ERK1/2 as a biomarker in drug testing on CRC cell lines need to be re‐evaluated in light of these findings. We suggest SOX9 to be a more suitable biomarker although more biological insight of its role in CRC is needed. In the perspective of targeted therapy, we are of the opinion that inhibition of MAPK/PI3K signaling in CRC is unlikely to be effective. Moreover, the high phospho‐ERK1/2 in colon mucosa from NI‐WB implies that MAPK signaling possibly serve important functions in normal colonic homeostasis, thus inhibition of MAPK signaling may lead to undesirable consequences. A clearer insight into signaling in human CRC is essential for future therapeutic opportunities.

## Conflict of interest

The authors declare no conflict of interest.

## Data accessibility

The data generated in this study are available in Supporting Information Figs. S1–S3, S5. The analyses from public domain data are available in Supporting Information Figs. S4 and S6, Tables S4–S8. These data were derived from the following resources available in the NCBI GEO at https://www.ncbi.nlm.nih.gov/geo/ series GSE95132 [24], TCGA Research Network at https://www.cancer.gov/tcga via GDC at https://portal.gdc.cancer.gov or cBioPortal at https://www.cbioportal.org [22, 23], and CPTAC COAD dataset downloaded via LinkedOmics portal at http://linkedomics.org [21].

## Author contributions

PYC and SHW contributed to the concept, study design, and interpretation of the data. KKL contributed to the acquisition, analysis, and interpretation of the data. CLT and ET contributed to the tissue collection, clinical data, and administration. KKL and PYC drafted the manuscript. KKL, PYC, and SHW contributed to the critical revision of the manuscript. All authors read and approved the final manuscript.

## Supporting information


**Fig. S1**. NI‐WB of phospho‐ERK1/2 and total‐ERK1/2 for all 33 matched CRC mucosa (M) and tumor (T) pairs.
**Fig. S2**. NI‐WB of phospho‐Akt1/2/3 and pan‐Akt1/2/3 for all 33 matched CRC mucosa (M) and tumor (T) pairs.
**Fig. S3**. NI‐WB of SOX9 and ACTB for all 33 matched CRC mucosa (M) and tumor (T) pairs.
**Fig. S4**. Phospho‐ERK1/2 is downregulated in tumor compared to matched mucosa in the CPTAC‐COAD phosphoproteome dataset.
**Fig. S5**. Histological structure of matched mucosa with tumors of low or high phospho‐ERK1/2.
**Fig. S6**. SOX9 protein upregulation in CRC tumors vs matched mucosa from CPTAC‐COAD and *SOX9* mRNA upregulation in KRAS mutant vs WT CRC tumors from TCGA Pan Cancer Atlas.
**Table S1**. Clinico‐pathological characteristics of fresh frozen tumor and matched mucosa samples.
**Table S2**. Primers used for PCR amplification and sequencing of KRAS.
**Table S3**. Primary and secondary antibodies and their dilutions in NI‐WB.
**Table S4**. Number of CRC tumor‐matched mucosa pairs and their respective *KRAS* and *BRAF* mutation status retrieved from TCGA and CPTAC.
**Table S5**. The number of upregulated and downregulated genes from the TCGA and NCBI GEO series GSE95132 datasets mapped in DAVID and g:Profiler.
**Table S6**. MAPK‐related GO : BP, KEGG and REACTOME terms associated with upregulated (A) and downregulated (B) genes in both TCGA (*KRAS* WT and mutant Tumors vs Matched Mucosa) and NCBI GEO series GSE95132 (Tumour vs Mucosa and ACFs vs NCs).
**Table S7**. PI3K/Akt‐related GO : BP, KEGG and REACTOME terms associated with upregulated genes (A) and downregulated genes (B) in both TCGA (*KRAS* WT and mutant tumors vs matched mucosa) and NCBI GEO series GSE95132 (Tumour vs Mucosa and ACFs vs NCs).
**Table S8**. Differential Expression of *SOX9* from TCGA and NCBI GEO series GSE95132 transcriptome data of tumor compared to matched mucosa, or aberrant crypt foci compared to normal crypts.
