# Supplementary material for: KRAS mutation‐independent downregulation of MAPK/PI3K signaling in colorectal cancer
Source: Mol Oncol. 2022 Jan 1;16(5):1171–83. doi: 10.1002/1878-0261.13163 (PMC8895447; doi:10.1002/1878-0261.13163)
Supplement: Supplementary file 1 — Fig. S1. NI‐WB of phospho‐ERK1/2 and total‐ERK1/2 for all 33 matched CRC mucosa (M) and tumor (T) pairs. Fig. S2. NI‐WB of phospho‐Akt1/2/3 and pan‐Akt1/2/3 for all 33 matched CRC mucosa (M) and tumor (T) pairs. Fig. S3. NI‐WB of SOX9 and ACTB for all 33 matched CRC mucosa (M) and tumor (T) pairs. Fig. S4. Phospho‐ERK1/2 is downregulated in tumor compared to matched mucosa in the CPTAC‐COAD phosphoproteome dataset. Fig. S5. Histological structure of matched mucosa with tumors of low or high phospho‐ERK1/2. Fig. S6. SOX9 protein upregulation in CRC tumors vs matched mucosa from CPTAC‐COAD and SOX9 mRNA upregulation in KRAS mutant vs WT CRC tumors from TCGA Pan Cancer Atlas. Table S1. Clinico‐pathological characteristics of fresh frozen tumor and matched mucosa samples. Table S2. Primers used for PCR amplification and sequencing of KRAS. Table S3. Primary and secondary antibodies and their dilutions in NI‐WB. Table S4. Number of CRC tumor‐matched mucosa pairs and their respective KRAS and BRAF mutation status retrieved from TCGA and CPTAC. Table S5. The number of upregulated and downregulated genes from the TCGA and NCBI GEO series GSE95132 datasets mapped in DAVID and g:Profiler. Table S6. MAPK‐related GO : BP, KEGG and REACTOME terms associated with upregulated (A) and downregulated (B) genes in both TCGA (KRAS WT and mutant Tumors vs Matched Mucosa) and NCBI GEO series GSE95132 (Tumour vs Mucosa and ACFs vs NCs). Table S7. PI3K/Akt‐related GO : BP, KEGG and REACTOME terms associated with upregulated genes (A) and downregulated genes (B) in both TCGA (KRAS WT and mutant tumors vs matched mucosa) and NCBI GEO series GSE95132 (Tumour vs Mucosa and ACFs vs NCs). Table S8. Differential Expression of SOX9 from TCGA and NCBI GEO series GSE95132 transcriptome data of tumor compared to matched mucosa, or aberrant crypt foci compared to normal crypts. [file MOL2-16-1171-s001.docx]

***KRAS* mutation-independent downregulation of MAPK/PI3K signaling in colorectal cancer**

**Kuen Kuen Lam, Choong Leong Tang, Emile Tan, Siew Heng Wong, Peh Yean Cheah**

**Supporting Information**

**Supplementary Figures**

**Figure S1. NI-WB of phospho-ERK1/2 and total-ERK1/2 for all 33 matched CRC mucosa (M) and tumor (T) pairs.** The patient number is indicated above each M/T pair, and the KRAS mutation status of the tumor is indicated above the patient number. The upper 44 kDa band is phospho-ERK1 / total ERK1 and the lower 42 kDa band is phospho-ERK2 / total ERK2. * Sample from patient 5482 was repeated. p-ERK1/2, phospho-ERK1/2; tERK1/2, total ERK1/2.

**Figure S2. NI-WB of phospho-Akt1/2/3 and pan-Akt1/2/3 for all 33 matched CRC mucosa (M) and tumor (T) pairs.** The patient number is indicated above each M/T pair, and the KRAS mutation status of the tumor is indicated above the patient number. Both phospho-Akt1/2/3 and pan-Akt migrates at ~ 60 kDa. * Sample 7098 was omitted due to degradation of mucosa sample. p-Akt1/2/3, phospho-Akt1/2/3.

**Figure S3. NI-WB of SOX9 and ACTB for all 33 matched CRC mucosa (M) and tumor (T) pairs.** The patient number is indicated above each M/T pair, and the KRAS mutation status of the tumor is indicated above the patient number. The SOX9 band migrates at ~75 kDa although its molecular weight is 56 kDa, the ACTB band migrates at ~45 kDa.* Samples 5479 and 4569 was omitted due to degradation.

**Figure S4.** **Phospho-ERK1/2 is downregulated in tumor compared to matched mucosa in the CPTAC-COAD phosphoproteome dataset.** Box and whiskers plot of log_2_FC values of tumor vs mucosa of phospho-ERK1(Y204) and phospho-ERK2(Y187) from CPTAC-COAD phosphoproteome dataset [1]. The dotted line indicates log_2_FC=0. Sample size for KRAS WT (n=46), G12D (n=11), G12V (n=7), G13D (n=3) and Others (n=13), BRAF V600E (n=12) and others (n=4). All BRAF V600E are KRAS WT. p-ERK1, phospho-ERK1; p-ERK2, phospho-ERK2. * one sample two-tailed t-test, H_0_: log_2_FC = 0, p<0.05.

**Figure S5. Histological structure of matched mucosa with tumors of low or high phospho-ERK1/2.** Toluidine blue stained cryosections of matched mucosa (A, C) and tumors (B, D) with lower phospho-ERK1/2 (T/M=0.6) in tumor (B) and higher phospho-ERK1/2 (T/M=1.5) in tumor (D) than mucosa. Both tumors harbor KRAS G12V mutations. Scale bar = 200 µm in (A) is applicable to all images.

**Figure S6. SOX9 protein upregulation in CRC tumors vs matched mucosa from CPTAC-COAD and *SOX9* mRNA upregulation in *KRAS* mutant vs WT CRC tumors from TCGA Pan Cancer Atlas.** Scatter plot with median line of SOX9 protein levels log_2_FC (tumor vs mucosa) from CPTAC-COAD proteome dataset (A) and *SOX9* mRNA levels of CRC tumor samples from TCGA Pan Cancer Atlas dataset retrieved via cBioPortal (<https://www.cbioportal.org/>) (B), categorized by individual *KRAS* and *BRAF* mutations [1-3]. In (A), all *BRAF* mutated tumor are *KRAS* WT. The dotted line indicates log_2_FC=0. Total sample size n=75: *KRAS* WT (n=33), G12D (n=10), G12V (n=6), G13D (n=3) and Others (n=11), *BRAF* V600E (n=8) and others (n=4). In (B), *SOX9* mRNA levels are represented by RSEM (RNA-Seq by Expectation-Maximization) values which is batch normalized from Illumina RNASeqV2. Total sample size n=435: *KRAS* WT (n=230), G12D (n=46), G12V (n=46), G13D (n=29) and Others (n=63), *BRAF* V600E (n=9) and others (n=12). * Unpaired one-tailed t-test with Welch’s correction, p<0.05; ** unpaired two-tailed t-test with Welch’s correction, p<0.05; Δ, one-sample t-test H_0_: log_2_FC=0, p<0.05.

**Table S1**. Clinico-pathological characteristics of fresh frozen tumor and matched mucosa samples.

|  | **Test Samples (n=33)** | |
| --- | --- | --- |
| **Characteristics** | **No. of patients** | **%** |
| **Age, years** (median = 70) |  |  |
| ≤70 | 20 | 60.6 |
| >70 | 13 | 39.4 |
|  |  |  |
| **Gender** |  |  |
| Male | 16 | 48.5 |
| Female | 17 | 51.5 |
|  |  |  |
| **Tumor Site** |  |  |
| Right | 13 | 39.4 |
| Left | 20 | 60.6 |
|  |  |  |
| **Duke's stage** |  |  |
| A | 4 | 12.1 |
| B | 9 | 27.3 |
| C | 19 | 57.6 |
| D | 1 | 3.0 |
|  |  |  |
| **Differentiation** |  |  |
| Well differentiated | 4 | 12.1 |
| Moderately differentiated | 26 | 78.8 |
| Poorly differentiated | 1 | 3.0 |
| Mucinous | 1 | 3.0 |

**Table S2.** Primers used for PCR amplification and sequencing of KRAS

| **Target** | **Primer** | **Sequence** |
| --- | --- | --- |
| KRAS Exon 2 | KRAS Ex2(-185)F*  KRAS Ex2(+198)R | TCATTACGATACACGTCTGCAG  CCCTGACATACTCCCAAGG |
| KRAS Exon 3 | KRAS Ex3(-169)F*  KRAS Ex3(+173)R | TTCAGGTGCTTAGTGGCC  CACTGCTCTAATCCCCCAAG |
| KRAS Exon 4 | KRAS Ex4(-208)F*  KRAS Ex4(+175)R | TTGATCTTTTGAGAGAGATACAAGG  GCAGTACCATGGACACTGG |

*These primers are used for Sanger sequencing reaction.

**Table S3.** Primary and secondary antibodies and their dilutions in NI-WB.

| **Primary antibodies** | **Brand and catalogue number** | **Dilution** |
| --- | --- | --- |
| mouse anti-ERK1/2 | Santa Cruz Biotechnology, sc-514302 | 1:800 |
| rabbit anti phospho-ERK1/2(T202/Y204) | Cell Signalling Technology, 4370 | 1:2000 |
| mouse anti-Akt (pan) (40D4) | Cell Signalling Technology, 2920 | 1:2000 |
| rabbit anti-p-Akt(S473) | Cell Signalling Technology, 9271 | 1:1000 |
| rabbit anti-SOX9 | Merck, AB5535 | 1:2000 |
| mouse anti-ACTB | Proteintech, 60008-1-Ig | 1:20,000 |
| **Secondary antibodies** |  |  |
| 800CW IRDye® donkey anti-mouse IgG | LI-COR Biosciences, 925-32212 | 1:20,000 |
| 680RD IRDye® donkey anti-rabbit IgG | LI-COR Biosciences, 925-68073 | 1:20,000 |

**Table S4.** Number of CRC tumor-matched mucosa pairs and their respective *KRAS* and *BRAF* mutation status retrieved from TCGA and CPTAC [1].

|  | ***KRAS*** | | | | |  | ***BRAF*** | |
| --- | --- | --- | --- | --- | --- | --- | --- | --- |
|  | **WT** | **G12D** | **G12V** | **G13D** | **Others** |  | **WT** | **V600E** |
| **TCGA Transcriptome** | 24 | 2 | 3 | 3 | 3 |  | - | - |
|  |  |  |  |  |  |  |  |  |
| **CPTAC Phosphoproteome**  p-ERK1(Y204) / p-ERK2(Y187)* | 46 | 11 | 7 | 3 | 13 |  | 12 | 4 |
|  |  |  |  |  |  |  |  |  |
| **CPTAC Proteome**  SOX9 | 35 | 10 | 6 | 3 | 11 |  | 8 | 4 |

CPTAC samples without numerical data were omitted. *p-ERK=phospho-ERK

**Table S5.** The number of upregulated and downregulated genes from the TCGA and NCBI GEO series GSE95132 [4] datasets mapped in DAVID [5, 6] and g:Profiler [7, 8].

|  | **Upregulated genes** | |  | **Downregulated genes** | |
| --- | --- | --- | --- | --- | --- |
| **Datasets** | **DAVID** | **g:Profiler** |  | **DAVID** | **g:Profiler** |
| TCGA KRAS WT (24 pairs)^a,c^ | 4619 | 4884 |  | 4274 | 4505 |
| TCGA KRAS mut (11 pairs)^a,c^ | 3620 | 3826 |  | 3577 | 3756 |
| GSE95132 (tissue, 10 pairs)^a,d^ | 64 | 59 |  | 230 | 191 |
| GSE95132 (crypts, 5 pairs)^b,d^ | 39 | 40 |  | 24 | 25 |

^a^ For TCGA *KRAS* WT and mut, and GSE95312-tissue dataset, comparison was done between tumor and mucosa, the latter is taken as reference.

^b^ For GSE95132-crypts dataset, comparison was performed between ACF vs NCs, the latter is taken as reference.

^c^ For both the TCGA *KRAS* WT and mut datasets, only significantly differentially expressed genes with fold-change ≥ 1.5 were inputted into DAVID and g:Profiler.

^d^ For GSE95132, all significantly differentially expressed genes were inputted into DAVID and g:Profiler.

**Table S6.** MAPK-related GO:BP, KEGG and REACTOME terms associated with upregulated (A) and downregulated (B) genes in both TCGA (*KRAS* WT and mutant Tumors vs Matched Mucosa) and NCBI GEO series GSE95132 [4] (Tumour vs Mucosa and ACFs vs NCs). The mapping was performed by DAVID [5, 6] and g:Profiler [7, 8].

| **A** | **P-value** | | | | | | | | |
| --- | --- | --- | --- | --- | --- | --- | --- | --- | --- |
|  | **DAVID*** | | | |  | **g:Profiler*** | | | |
|  | **TCGA** | | **GSE95132** | |  | **TCGA** | | **GSE95132** | |
| **GO:BP terms** | **T_K-WT_/M** | **T_K-mut_/M** | **T/M** | **ACF/NCs** |  | **T_K-WT_/M** | **T_K-mut_/M** | **T/M** | **ACF/NCs** |
| GO:0000165~MAPK cascade | 0.995 | 1.000 | 1 | 1 |  | 1 | 1 | 1 | 1 |
| GO:0043408~regulation of MAPK cascade | 0.623 | 0.706 | - | - |  | 1 | 1 | - | - |
| GO:0043410~positive regulation of MAPK cascade | 0.091 | 0.942 | - | - |  | 1 | 1 | - | - |
| GO:0043409~negative regulation of MAPK cascade | 1 | 0.923 | - | - |  | 1 | 1 | - | - |
| GO:0000187~activation of MAPK activity | 0.857 | 0.636 | - | - |  | 1 | 1 | - | - |
| GO:0000186~activation of MAPKK activity | 0.998 | 0.962 | - | - |  | 1 | 1 | - | - |
| GO:0000188~inactivation of MAPK activity | 0.239 | 0.411 | - | - |  | 1 | 1 | - | - |
|  |  |  |  |  |  |  |  |  |  |
| **KEGG terms** |  |  |  |  |  |  |  |  |  |
| hsa04010:MAPK signaling pathway | 0.969 | 0.926 | 1 | - |  | 1 | 1 | 1 | - |
|  |  |  |  |  |  |  |  |  |  |
| **REACTOME terms** |  |  |  |  |  |  |  |  |  |
| R-HSA-5675221:Negative regulation of MAPK pathway | 0.834 | 0.969 | - | - |  | 1 | 1 | - | - |
| R-HSA-5674135:MAP2K and MAPK activation | 0.941 | 0.995 | - | - |  | 1 | 1 | - | - |
| R-HSA-112409:RAF-independent MAPK1/3 activation | 0.279 | 0.388 | - | - |  | 1 | 1 | - | - |
| R-HSA-198753:ERK/MAPK targets | 1 | 1 | - | - |  | 1 | 1 | - | - |
| R-HSA-110056:MAPK3 (ERK1) activation | 0.649 | 0.847 | - | - |  | 1 | 1 | - | - |
| R-HSA-112411:MAPK1 (ERK2) activation | 0.878 | 1 | - | - |  | 1 | 1 | - | - |

| **B** | **P-Value** | | | | | | | | |
| --- | --- | --- | --- | --- | --- | --- | --- | --- | --- |
|  | **DAVID*** | | | |  | **g:Profiler*** | | |  |
|  | **TCGA** | | **GSE95132** | |  | **TCGA** | | **GSE95132#** | |
| **GO:BP terms** | **T_K-WT_/M** | **T_K-mut_/M** | **T/M** | **ACF/NCs** |  | **T_K-WT_/M** | **T_K-mut_/M** | **T/M** | |
| GO:0043410~positive regulation of MAPK cascade | **0.005** | **1.44E-04** | 1 | - |  | **2.08E-07** | **2.40E-07** | 1 | |
| GO:0000165~MAPK cascade | **0.006** | **0.038** | 0.863 | - |  | **1.89E-06** | **3.82E-05** | 1 | |
| GO:0070374~positive regulation of ERK1 and ERK2 cascade | - | **3.08E-04** | 0.379 | - |  |  | 0.014 | 1 | |
| GO:0000187~activation of MAPK activity | **0.037** | **0.025** | 1 | - |  | 1 | 1 | 1 | |
| GO:0070372~regulation of ERK1 and ERK2 cascade |  | 0.654 | - | - |  |  | 0.042 | - | |
| GO:0043408~regulation of MAPK cascade | 0.621 | 0.740 | 1 | - |  | **1.77E-07** | **2.77E-07** | 1 | |
| GO:0000185~activation of MAPKKK activity | 0.691 | 0.878 | - | - |  | 1 | 1 | - | |
|  | - | - | - | - |  |  | - | - | |
| **KEGG terms** | - | - | - | - |  |  | - | - | |
| hsa04010:MAPK signaling pathway | **0.015** | 0.094 | 1 | - |  | 0.818 | 1 | 1 | |
|  | - | - | - | - |  |  | - | - | |
| **REACTOME terms** | - | - | - | - |  |  | - | - | |
| R-HSA-198753:ERK/MAPK targets | 0.249 | 0.350 | - | - |  | 1 | 1 | - | |
| R-HSA-5674135:MAP2K and MAPK activation | 0.280 | 0.939 | - | - |  | 1 | 1 | - | |
| R-HSA-110056:MAPK3 (ERK1) activation | 0.329 | 0.856 | - | - |  | 1 | 1 | - | |
| R-HSA-112411:MAPK1 (ERK2) activation | 0.571 | 0.825 | - | - |  | 1 | 1 | - | |

*Output from DAVID uses Modified Fisher Exact adjusted P-value and output from g:Profiler uses tailor-made algorithm g:SCS adjusted p-value.

Bold number indicates significant p-values (p<0.05).

# GSE95132 ACFs/NCs downregulated genes is not associated with any MAPK-related GO:BP, KEGG and REACTOME terms hence not included in this table.

ACF, aberrant crypt foci; M, matched mucosa; NCs, normal crypts. T, tumor.T_K-WT_, tumors with WT *KRAS*; T_K-mut,_ tumors with mutant *KRAS*.

**Table S7.** PI3K/Akt-related GO:BP, KEGG and REACTOME terms associated with upregulated genes (A) and downregulated genes (B) in both TCGA (*KRAS* WT and mutant tumors vs matched mucosa) and NCBI GEO series GSE95132 [4] (Tumour vs Mucosa and ACFs vs NCs). The mapping was performed by DAVID [5, 6] and g:Profiler [7, 8].

| **A** | **P-value** | | | | | | | | |
| --- | --- | --- | --- | --- | --- | --- | --- | --- | --- |
|  | **DAVID*** | | | |  | **g:Profiler*** | | | |
|  | **TCGA** | | **GSE95132** | |  | **TCGA** | | **GSE95132** | |
| **KEGG terms** | **T_K-WT_/M** | **T_K-mut_/M** | **T/M** | **ACF/NCs** |  | **T_K-WT_/M** | **T_K-mut_/M** | **T/M** | **ACF/NCs** |
| hsa04151:PI3K-Akt signaling pathway | 0.545 | 0.402 | 1 | 1 |  | 1 | 1 | 1 | 1 |
| **REACTOME terms** |  |  |  |  |  |  |  |  |  |
| R-HSA-1257604:PIP3 activates AKT signaling | 0.739 | 0.950 | 1 | - |  | 1 | 1 | 1 | 1 |
| R-HSA-199418:Negative regulation of the PI3K/AKT network | 0.846 | 1 | 1 | - |  | 1 | 1 | 1 | - |
| R-HSA-2219530:Constitutive Signaling by Aberrant PI3K in Cancer | 0.753 | 0.916 | - | 1 |  | 1 | 1 | - | 1 |
| R-HSA-109704:PI3K Cascade | 0.485 | 0.761 | - | 1 |  | 1 | 1 | 1 | 1 |
| R-HSA-5674400:Constitutive Signaling by AKT1 E17K in Cancer | 0.978 | - | - | - |  | 1 | - | - | - |
| R-HSA-392451:G beta:gamma signalling through PI3Kgamma | 0.953 | 0.999 | - | - |  | 1 | 1 | - | - |
| R-HSA-389357:CD28 dependent PI3K/Akt signaling | 0.703 | 1 | 1 | - |  | 1 | 1 | 1 | - |
| R-HSA-198323:AKT phosphorylates targets in the cytosol | 0.952 | - | - | - |  | 1 | - | - | - |
| R-HSA-1963642:PI3K events in ERBB2 signaling | 1 | 0.950 | - | - |  | 1 | 1 | - | - |
| R-HSA-198693:AKT phosphorylates targets in the nucleus | 1 | - | - | - |  | 1 | - | - | - |
| R-HSA-1250342:PI3K events in ERBB4 signaling | 1 | 1 | - | - |  | 1 | 1 | - | - |
| R-HSA-198203:PI3K/AKT activation | 0.878 | - | - | 1 |  | 1 | - | - | 1 |
| R-HSA-211163:AKT-mediated inactivation of FOXO1A | 1 | - | - | - |  | 1 | - | - | - |

| **B** | **P-value** | | | | | | |  |
| --- | --- | --- | --- | --- | --- | --- | --- | --- |
|  | **DAVID*** | | |  | **g:Profiler*** | | |  |
|  | **TCGA** | | **GSE95132^#^** |  | **TCGA** | | **GSE95132^#^** |  |
| **KEGG terms** | **T_K-WT_/M** | **T_K-mut_/M** | **T/M** |  | **T_K-WT_/M** | **T_K-mut_/M** | **T/M** |  |
| hsa04151:PI3K-Akt signaling pathway | **0.007** | **0.106** | 0.50084 |  | 0.652 | 1 | 1 |  |
| **REACTOME terms** |  |  |  |  |  |  |  |  |
| R-HSA-1257604:PIP3 activates AKT signaling | **0.004** | **0.021** | 1 |  | 1 | 1 | 1 |  |
| R-HSA-199418:Negative regulation of the PI3K/AKT network | 0.503 | 1 | - |  | 0.528 | 1 | - |  |
| R-HSA-2219530:Constitutive Signaling by Aberrant PI3K in Cancer | **1.64E-04** | **4.30E-04** | 1 |  | 0.383 | 0.938 | 1 |  |
| R-HSA-109704:PI3K Cascade | 0.176 | 0.452 | 1 |  | 1 | 1 | 1 |  |
| R-HSA-5674400:Constitutive Signaling by AKT1 E17K in Cancer | 0.775 | 0.950 | - |  | 1 | 1 | - |  |
| R-HSA-392451:G beta:gamma signalling through PI3Kgamma | **0.004** | **0.033** | - |  | 0.999 | 1 | - |  |
| R-HSA-389357:CD28 dependent PI3K/Akt signaling | 0.993 | 1 | - |  | 1 | 1 | - |  |
| R-HSA-198323:AKT phosphorylates targets in the cytosol | 0.772 | 0.919 | - |  | 1 | 1 | - |  |
| R-HSA-1963642:PI3K events in ERBB2 signaling | **0.007** | **0.048** | - |  | 0.776 | 1 | - |  |
| R-HSA-198693:AKT phosphorylates targets in the nucleus | 0.571 | 1 | - |  | 1 | 1 | - |  |
| R-HSA-1250342:PI3K events in ERBB4 signaling | **0.034** | **0.019** | - |  | 1 | 1 | - |  |
| R-HSA-198203:PI3K/AKT activation | 1 | - | - |  | 1 | - | - |  |
| R-HSA-211163:AKT-mediated inactivation of FOXO1A | 0.595 | 1 | - |  | 1 | 1 | - |  |

*Output from DAVID uses Modified Fisher Exact adjusted P-value and output from g:Profiler uses tailor-made algorithm g:SCS adjusted p-value.

Bold number indicates significant p-values (p<0.05).

# GSE95132 ACFs/NCs downregulated genes is not associated with any PI3K/Akt-related GO:BP, KEGG and REACTOME terms hence not included in this table.

ACF, aberrant crypt foci; M, matched mucosa; NCs, normal crypts. T, tumor.T_K-WT_, tumors with WT *KRAS*; T_K-mut,_ tumors with mutant *KRAS*.

**Table S8.** Differential Expression of *SOX9* from TCGA and GSE95132 transcriptome data of tumor compared to matched mucosa, or aberrant crypt foci compared to normal crypts.

|  | **TCGA** | |  | **GSE95132** | |
| --- | --- | --- | --- | --- | --- |
|  | **T_K-WT_/M** | **T_K-mut_/M** |  | **T/M** | **ACF/NCs** |
| **Fold Change** | 4.93 | 3.73 |  | 6.96 | 1.31 |
| **Adjusted p-value*** | **1.85E-21** | **2.92E-10** |  | **0.048** | 0.893 |

*The p-values were adjusted for multiple testing with the Benjamini-Hochberg procedure.

The differential expression values were derived from analysis using DESeq2 algorithm.

ACF, aberrant crypt foci; M, matched mucosa; NCs, normal crypts. T, tumor.T_K-WT_, tumors with WT *KRAS*; T_K-mut,_ tumors with mutant *KRAS*.

**References**

1. Vasaikar S, Huang C, Wang X, Petyuk VA, Savage SR, Wen B, Dou Y, Zhang Y, Shi Z, Arshad OA, Gritsenko MA, Zimmerman LJ, McDermott JE, Clauss TR, Moore RJ, Zhao R, Monroe ME, Wang YT, Chambers MC, Slebos RJC, Lau KS, Mo Q, Ding L, Ellis M, Thiagarajan M, Kinsinger CR, Rodriguez H, Smith RD, Rodland KD, Liebler DC, Liu T, Zhang B & Clinical Proteomic Tumor Analysis C (2019) Proteogenomic Analysis of Human Colon Cancer Reveals New Therapeutic Opportunities. Cell 177, 1035-1049 e19.

2. Cerami E, Gao J, Dogrusoz U, Gross BE, Sumer SO, Aksoy BA, Jacobsen A, Byrne CJ, Heuer ML, Larsson E, Antipin Y, Reva B, Goldberg AP, Sander C & Schultz N (2012) The cBio cancer genomics portal: an open platform for exploring multidimensional cancer genomics data. Cancer Discov 2, 401-4.

3. Gao J, Aksoy BA, Dogrusoz U, Dresdner G, Gross B, Sumer SO, Sun Y, Jacobsen A, Sinha R, Larsson E, Cerami E, Sander C & Schultz N (2013) Integrative analysis of complex cancer genomics and clinical profiles using the cBioPortal. Sci Signal 6, pl1.

4. Hanley MP, Hahn MA, Li AX, Wu X, Lin J, Wang J, Choi AH, Ouyang Z, Fong Y, Pfeifer GP, Devers TJ & Rosenberg DW (2017) Genome-wide DNA methylation profiling reveals cancer-associated changes within early colonic neoplasia. Oncogene 36, 5035-5044.

5. Huang DW, Sherman BT & Lempicki RA (2009) Bioinformatics enrichment tools: paths toward the comprehensive functional analysis of large gene lists. Nucleic Acids Res 37, 1-13.

6. Huang DW, Sherman BT & Lempicki RA (2009) Systematic and integrative analysis of large gene lists using DAVID bioinformatics resources. Nat Protoc 4, 44-57.

7. Reimand J, Kull M, Peterson H, Hansen J & Vilo J (2007) g:Profiler--a web-based toolset for functional profiling of gene lists from large-scale experiments. Nucleic Acids Res 35, W193-200.

8. Raudvere U, Kolberg L, Kuzmin I, Arak T, Adler P, Peterson H & Vilo J (2019) g:Profiler: a web server for functional enrichment analysis and conversions of gene lists (2019 update). Nucleic Acids Res 47, W191-W198.
